# Identification of highly variable sequence fragments in unmapped reads for rapid bacterial genotyping

**DOI:** 10.1186/s12864-022-08550-4

**Published:** 2022-12-29

**Authors:** Marketa Nykrynova, Vojtech Barton, Matej Bezdicek, Martina Lengerova, Helena Skutkova

**Affiliations:** 1 Department of Biomedical Engineering, Faculty of Electrical Engineering and Communication, Brno University of Technology, Brno, Czechia; 2 Department of Internal Medicine, Hematology and Oncology, University Hospital Brno, Brno, Czechia

**Keywords:** Bacterial genotyping, Genome assembly, Unmapped reads, De novo assembly, Multilocus sequence typing, Mini-MLST

## Abstract

**Background:**

Bacterial genotyping is a crucial process in outbreak investigation and epidemiological studies. Several typing methods such as pulsed-field gel electrophoresis, multilocus sequence typing (MLST) and whole genome sequencing are currently used in routine clinical practice. However, these methods are costly, time-consuming and have high computational demands. An alternative to these methods is mini-MLST, a quick, cost-effective and robust method based on high-resolution melting analysis. Nevertheless, no standardized approach to identify markers suitable for mini-MLST exists. Here, we present a pipeline for variable fragment detection in unmapped reads based on a modified hybrid assembly approach using data from one sequencing platform.

**Results:**

In routine assembly against the reference sequence, high variable reads are not aligned and remain unmapped. If de novo assembly of them is performed, variable genomic regions can be located in created scaffolds. Based on the variability rates calculation, it is possible to find a highly variable region with the same discriminatory power as seven housekeeping gene fragments used in MLST. In the work presented here, we show the capability of identifying one variable fragment in de novo assembled scaffolds of 21 *Escherichia coli* genomes and three variable regions in scaffolds of 31 *Klebsiella pneumoniae* genomes. For each identified fragment, the melting temperatures are calculated based on the nearest neighbor method to verify the mini-MLST’s discriminatory power.

**Conclusions:**

A pipeline for a modified hybrid assembly approach consisting of reference-based mapping and de novo assembly of unmapped reads is presented. This approach can be employed for the identification of highly variable genomic fragments in unmapped reads. The identified variable regions can then be used in efficient laboratory methods for bacterial typing such as mini-MLST with high discriminatory power, fully replacing expensive methods such as MLST. The results can and will be delivered in a shorter time, which allows immediate and fast infection monitoring in clinical practice.

**Supplementary Information:**

The online version contains supplementary material available at (10.1186/s12864-022-08550-4).

## Background

Bacterial genotyping is a powerful tool to investigate the relationships between individual strains from a single species as well as to study the bacterial population structure and dynamics. Phenotypic and genotypic methods can be applied to distinguish bacteria, with genotyping most often being used these days [1].

For a long time, the method considered a ’gold standard’ for bacterial genotyping in routine practice was pulsed-field gel electrophoresis, where DNA banding patterns are analyzed [2]. The main advantage of this method is its discriminatory power and intra-laboratory reproducibility, but on the other hand, it is time and labor intensive [3].

Another typing method used worldwide is multilocus sequence typing (MLST), a standardized and highly discriminatory technique. Several housekeeping genes’ 450 - 500 bp long fragments are sequenced and analyzed. For each allele of a gene, a unique number is assigned [4]. The combination of numbers for all the genes’ alleles define the allelic profiles represented by sequence types (STs). The MLST schemes are deposited in publicly available databases; thus, the method can be used for comparative epidemiological studies and monitoring the spread of high-risk strains. The MLST is a portable, standardized and reproducible method; however, the sequencing cost is still high. [5].

An alternative to MLST is mini-MLST, where the sequencing is replaced by high resolution melting analysis (HRM) [6]. In the first step, housekeeping gene fragments are amplified by PCR. Next, HRM is performed and as a result, a melting curve is obtained. When 50% of the DNA is denatured, the melting temperature is determined [7]. Each melting curve represents an individual melt allele named according to the GC base content in the amplified region. A combination of melt alleles from each gene defines the so-called melt type. The main advantage is the low cost, which is about 10 – 20% of MLST, low time demands and very high throughput [8]. However, there is no standardized approach to detect genetic markers suitable for mini-MLST, and often the sequences chosen for typing do not have sufficient discriminatory power.

The method with the highest discriminatory power for bacterial strain genotyping is whole genome sequencing [9]. Recently, genome sequencing has become more accessible to routine laboratories; however, the main bottlenecks in post-sequencing data analysis remain. The first bottleneck is the lack of standard protocols for data processing. This is because many tools exist, and each uses different data quality assessments, data processing, and results interpretation. The second bottleneck is in genome assembly, where the outputs are crucial for clinical practice. Two main approaches exist here: de novo assembly and reference-based assembly.

de novo assembly is time-consuming and computationally demanding, high-quality data are also required. High-throughput technologies provide short reads where the assembly is challenging, especially in repetitive regions [10]. As a result, the whole genome is not obtained, but only a large number of short contigs are generated [11]. These drawbacks make it difficult to use de novo assembly in routine clinical practice.

On the other hand, reference-based assembly is faster and less computationally demanding; nevertheless, the choice of an inappropriate reference sequence significantly affects the analyses and final results. In the reference assembly, only the shared genome parts are analyzed; however, the unmapped reads can contain important information, as the bacterial genomes are highly variable [12].

Hybrid assembly can be conducted to overcome de novo and reference-based assembly’s drawbacks. This approach combines two sequencing technologies and can be used to analyze unique genome parts [13]. However, this double-sequencing approach is not used as standard in clinical studies as it requires two sequencing platforms, which means extra cost and time.

Here we present a new approach based on reference-based mapping and de novo assembly, which can be compared to a hybrid assembly approach. The main difference is that data were obtained from only one sequencing platform, specifically Illumina Miseq, one of the most frequently used platforms in clinical practice [14]. Our goal is not to obtain a complete whole genome but only the most variable genomic regions that can be used in mini-MLST. The reference-based assembly is used as a filter to remove the low variable reads, which will map to the reference sequence. From the high variability reads that did not map to the reference, scaffolds are assembled. In the scaffolds, the most variable parts are identified, and these genomic regions can be used to distinguish bacteria in mini-MLST. Thus, more sequencing will not be necessary as further samples of the given bacterium will be classified based on mini-MLST analysis using the identified variable fragments.

In the present study, our goal was to analyze whole genome sequencing data obtained from isolates representing two clinically important bacterial species - *Escherichia coli* and *Klebsiella pneumoniae*. *E. coli* is a Gram-negative bacterium of the *Enterobacteriaceae* family [15], and includes pathogenic and commensal clones. Commensal clones are natural inhabitants of the human gastrointestinal tract and cause diseases only in immunocompromised patients or those with breached gastrointestinal barriers [16], [17]. Pathogenic strains can cause urinary tract infections, sepsis, or enteric diseases [15]. *E. coli* genome size varies from 4.2 to 6.0 Mb with an average of about 5 Mb [18]. *K. pneumoniae* is another bacterium from the *Enterobacteriaceae* family. It is an opportunistic pathogen that causes serious diseases such as pneumonia, bloodstream infections, urinary tract infections or sepsis, mainly in immunocompromised patients [19, 20]. In recent years, the number of antibiotic-resistant strains has increased [21]; thus, *K. pneumoniae* has become one of the major threats due to significant morbidity and mortality [8]. The genome has approximately 5.5 Mbp, and encodes about 5500 genes [22].

## Results and discussion

### Sequence type determination

The reference-based assembled consensus sequences were used for in silico MLST analysis. For *E. coli* the Warwick MLST scheme was employed; thus, seven housekeeping genes (*adk, fumC, gyrB, icd, mdh, puA, recA*) were analyzed. In total 11 sequence types were present in our dataset (1 x ST 69, 4 x ST 131, 1 x ST 95, 2 x ST 404, 2 x ST 38, 2 x ST 1049, 4 x ST 58, 1 x ST 297, 1 x ST 517, 2 x ST 101, 1 x ST UNW). The complete results of MLST typing are published in [23].

The sequence types of *K. pneumoniae* isolates were determined using seven housekeeping genes (*gapA, infB, mdh, pgi, phoE, rpoB, tonB*). Overall four STs were identified in the dataset (5 x ST 45, 9 x ST 405, 13 x ST 551, 4 x ST 950). The results of the MLST analysis are attached in Additional file 1: Table S1.

### Assembly analysis

The number of obtained reads for *E. coli* genomes were from 2 514 472 to 5 137 462, and for *K. pneumoniae*, the number varied from 1 608 984 to 4 001 068. In both datasets, less than 0.14% of reads mapped to the human genome; thus, no serious contamination occurred in the sequencing data. Trimmomatic removed about 5% of *E. coli* reads and approximately 13% of *K. pneumoniae* reads, and the remaining 95% and 87% from the total number of reads were used for further assembly. The number of reads mapped to the reference sequence varied from 64% to 81% for *E. coli* genomes and from 54% to 79% for *K. pneumoniae* genomes. Thus, around 17% of reads remained unmapped, on average. Complete information on the numbers of total, mapped, and unmapped reads is shown in the Tables 1 and 2.

**Table 1:** The numbers of all reads obtained from sequencing and number of mapped and unmapped reads to reference sequence for each *E. coli* genome

Genome id	Total number of reads	Number of reads mapped to reference sequence	Number of unmapped reads
EC155	4031662	2953193 (73.25%)	801054 (19.87%)
EC156	3544566	2367147 (66.78%)	906120 (25.56%)
EC157	3333908	2264411 (67.92%)	751002 (22.53%)
EC158	3984774	2983151 (74.86%)	683866 (17.16%)
EC159	3776174	2853515 (75.57%)	632261 (16.74%)
EC160	3112904	2351050 (75.53%)	593368 (19.06%)
EC161	3267268	2302509 (70.47%)	735359 (22.51%)
EC162	3572526	2300743 (64.40%)	915207 (25.62%)
EC163	3626826	2706501 (74.62%)	674798 (18.61%)
EC164	3332504	2221564 (66.66%)	840645 (25.23%)
EC165	3184082	2295010 (72.08%)	470745 (14.78%)
EC166	3070262	2057830 (67.02%)	807256 (26.29%)
EC167	5137462	3771148 (73.40%)	1062188 (20.68%)
EC168	3020940	2084303 (69.00%)	744089 (24.63%)
EC169	3277332	2277124 (69.48%)	767589 (23.42%)
EC170	3104926	2160099 (69.57%)	733623 (23.63%)
EC171	3122874	2536284 (81.22%)	400601 (12.83%)
EC172	2514472	2018011 (80.26%)	364826 (14.51%)
EC173	2930570	2326282 (79.38%)	409058 (13.96%)
EC174	2625002	2082053 (79.32%)	348544 (13.28%)
EC1773	2953930	2362794 (79.99%)	413730 (14.01%)

**Table 2:** The numbers of all reads obtained from sequencing and number of mapped and unmapped reads to reference sequence for each *K. pneumoniae* genome

Genome id	Total number of reads	Number of reads mapped to reference sequence	Number of unmapped reads
KP1179	3935088	3036116 (77.15%)	638924 (16.24%)
KP1182	4001068	3074207 (76.83%)	648782 (16.22%)
KP1183	1608984	1156116 (71.85%)	196156 (12.19%)
KP1188	3498596	2674209 (76.44%)	551172 (15.75%)
KP1193	3734192	2855397 (76.47%)	577781 (15.47%)
KP1196	3134684	2475800 (78.98%)	514079 (16.40%)
KP1205	2602914	1828407 (70.24%)	284748 (10.94%)
KP1214	2164256	1504126 (69.50%)	278202 (12.85%)
KP1215	2271372	1238457 (54.52%)	357472 (15.74%)
KP1216	2560420	1625530 (63.49%)	298045 (11.64%)
KP1217	2481622	1504278 (60.62%)	321015 (12.94%)
KP1225	2555570	1833621 (71.75%)	321722 (12.59%)
KP1226	3319880	2590238 (78.02%)	530031 (15.97%)
KP1231	3533000	2792224 (79.03%)	546440 (15.47%)
KP1235	2125072	1416555 (66.66%)	379393 (17.85%)
KP1237	1802568	1283958 (71.23%)	276591 (15.34%)
KP1238	3601626	2623619 (72.85%)	654699 (18.18%)
KP1241	3356164	2432489 (72.48%)	660648 (19.68%)
KP1251	2612190	1743842 (66.76%)	326689 (12.51%)
KP1252	2762088	1860343 (67.35%)	327610 (11.86%)
KP1255	2758768	1985975 (71.99%)	354107 (12.84%)
KP1256	2976516	2155608 (72.42%)	595617 (20.01%)
KP1257	3475000	2485612 (71.53%)	759590 (21.86%)
KP1258	3365802	2387789 (70.94%)	734237 (21.81%)
KP1261	3251662	2288750 (70.39%)	733627 (22.56%)
KP1262	3307022	2483487 (75.10%)	498093 (15.06%)
KP1263	2435788	1642209 (67.42%)	299499 (12.30%)
KP1267	2542032	1834260 (72.16%)	356682 (14.03%)
KP1268	2961438	1962571 (66.27%)	347947 (11.75%)
KP1269	3588114	2344679 (65.35%)	508768 (14.18%)
KP1273	3566426	2228144 (62.48%)	412178 (11.56%)

The unmapped reads of the 21 *E. coli* genomes and 31 *K. pneumoniae* genomes were de novo assembled via SPAdes. The number of scaffolds obtained for *E. coli* isolates varied from 151 to 296, and after removing the scaffolds shorter than 500 bp, the amount fluctuated from 124 to 230. In *K. pneumoniae* genomes were assembled from 86 to 323 scaffolds, and after removing short scaffolds, the number varied from 76 to 192. The largest scaffold lengths, N50 and L50, were determined and can be found in Tables 3 and 4 with other statistics. The detailed assembly process is described in the sections Reference-based assembly and De novo assembly of unmapped reads.

**Table 3:** Results of de novo analysis for *E. coli* genomes - number of assembled scaffolds, number of assembled scaffolds longer than 500 bp, length of the largest scaffold, N50 and L50 values

Genome ID	Number of scaffolds	Number of scaffolds longer than 500 bp	Largest scaffold	N50 for scaffolds longer than 500 bp	L50 for scaffolds longer than 500 bp
EC155	212	161	59711	10846	19
EC156	221	185	47494	10612	29
EC157	288	212	47492	9969	29
EC158	296	199	53287	12308	17
EC159	243	180	53348	12321	16
EC160	154	137	57276	15582	14
EC161	166	141	77372	25218	12
EC162	266	230	76720	11762	27
EC163	177	141	57271	17595	13
EC164	246	213	96303	11196	25
EC165	238	179	95072	7712	16
EC166	255	221	81897	11255	26
EC167	196	149	79395	18898	14
EC168	262	213	54793	11195	25
EC169	268	217	64992	14900	21
EC170	273	228	81897	10210	28
EC171	171	131	42845	9542	15
EC172	206	157	91847	10428	13
EC173	153	130	91182	11651	12
EC174	151	124	125059	18328	10
EC1773	224	162	62345	11505	14

**Table 4:** Results of de novo analysis for *K. pneumoniae* genomes - number of assembled scaffolds, number of assembled scaffolds longer than 500 bp, length of the largest scaffold, N50 and L50 values

Genome ID	Number of scaffolds	Number of scaffolds longer than 500 bp	Largest scaffold	N50 for scaffolds longer than 500 bp	L50 for scaffolds longer than 500 bp
KP1179	206	127	59157	16299	13
KP1182	268	147	68150	16519	13
KP1183	121	96	59451	16733	12
KP1188	142	102	60446	22213	10
KP1193	200	129	60144	21442	12
KP1196	166	109	59199	21305	12
KP1205	118	86	64806	22116	10
KP1214	120	94	51750	17243	13
KP1215	140	104	111137	19865	12
KP1216	109	89	63474	22220	11
KP1217	86	79	59905	30956	10
KP1225	95	76	73045	35831	8
KP1226	100	87	70022	21905	10
KP1231	130	95	70024	20544	11
KP1235	152	107	111131	20819	12
KP1237	109	87	44106	22101	12
KP1238	222	122	73045	17527	13
KP1241	323	192	53611	12228	17
KP1251	94	78	97605	22703	8
KP1252	97	82	63474	22636	9
KP1255	94	83	69812	21898	10
KP1256	224	135	53594	16189	14
KP1257	157	104	111195	19885	12
KP1258	150	103	111110	20820	11
KP1261	178	116	111110	20517	12
KP1262	191	115	70026	20540	11
KP1263	98	83	63478	22201	10
KP1267	96	83	80940	22120	9
KP1268	117	81	73010	22061	9
KP1269	182	120	73106	18185	12
KP1273	121	88	70026	21900	10

### Variable fragments identification

The scaffolds from genome EC162 and KP1241 were searched for in the remaining *E. coli* and *K. pneumoniae* genomes via BLAST+. These genomes were chosen as they contained the largest number of scaffolds. In total, from 230 scaffolds searched, only 25 scaffolds were located in all *E. coli* isolates and from 192 scaffolds, 78 were identified in all *K. pneumoniae* genomes.

The isolate sequences from corresponding scaffolds present in all genomes were aligned. Next, the most variable regions were located in the alignments, and in total, 11 variable fragments for *E. coli* and 244 fragments for *K. pneumoniae* were identified.

A phylogenetic tree was constructed for each variable fragment, and the number of clusters was determined (see Additional file 1: Table S2 and S3). From the obtained results, only one *E. coli* variable fragment (labelled as EC_01) was further analyzed as only this one could distinguish the isolates to 11 clusters, according to the MLST results. For *K. pneumoniae*, five variable fragments (labelled as KP_01, KP_02, KP_03, KP_04 and KP_05) classified the genomes correctly into four clusters according to their STs and were further analyzed. The analyzed variable fragments are shown in Table 5. The variable fragments’ identification is described in the section Detection of variable regions.

**Table 5:** The identified variable fragments located in assembled *E. coli* and *K. pneumoniae* genomes scaffolds with their size and number of clusters obtained by phylogenetic analysis

Name of variable fragment	Number of variable fragment	Variable fragment length [bp]	Number of clusters
EC_01	11	4762	11
KP_01	14	1346	4
KP_02	140	12	4
KP_03	146	13	4
KP_04	153	21	4
KP_05	154	26	4

### Variable fragment analyses

The variability calculation was used for the preliminary selection of sequences with higher variability. Before further analysis, Web BLAST was used to analyze the fragments and ensure that the sequences were from the bacterial chromosome and not from plasmids. In total, six variable fragments from both datasets were analyzed, and one *K. pneumoniae* fragment (KP_01) with a length of 1346 bp was removed as it contained only a plasmid sequence.

If the lengths of the identified fragments were long (more than 150 bp [7]), the sequences’ analysis via commonly used laboratory methods such as mini-MLST would be complicated. For this reason, the fragments can be shortened to identify the region with the highest variability rate. The process of shortening is described in the Fragment shortening section.

**Fig. 1:**
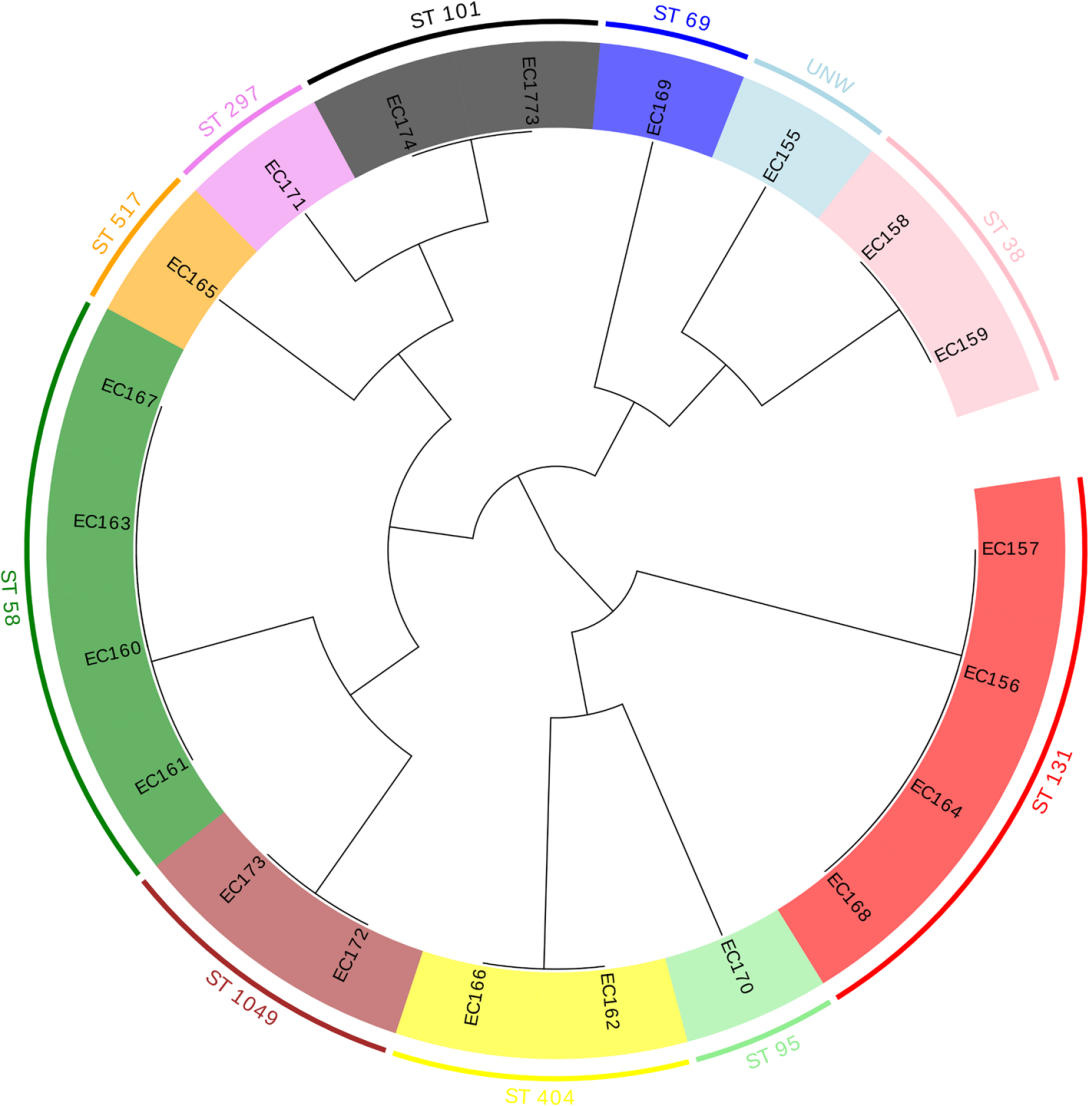
Cladogram of 21 *E. coli* isolates based on three variable fragments (EC_01_1, EC_01_2, EC_01_3) with highlight clusters obtained from MLST analysis created by Evolview [38]

The original length of the variable fragment (EC_01) with sufficient discriminatory power to distinguish *E. coli* genomes was 4,762 bp. After fragment erosion and decomposition, three fragments with a length of 120 bp were obtained (EC_01_1, EC_01_2, EC_01_3). The phylogenetic tree, constructed based on the three fragments with the highest variability, is depicted in Fig. 1.

**Fig. 2:**
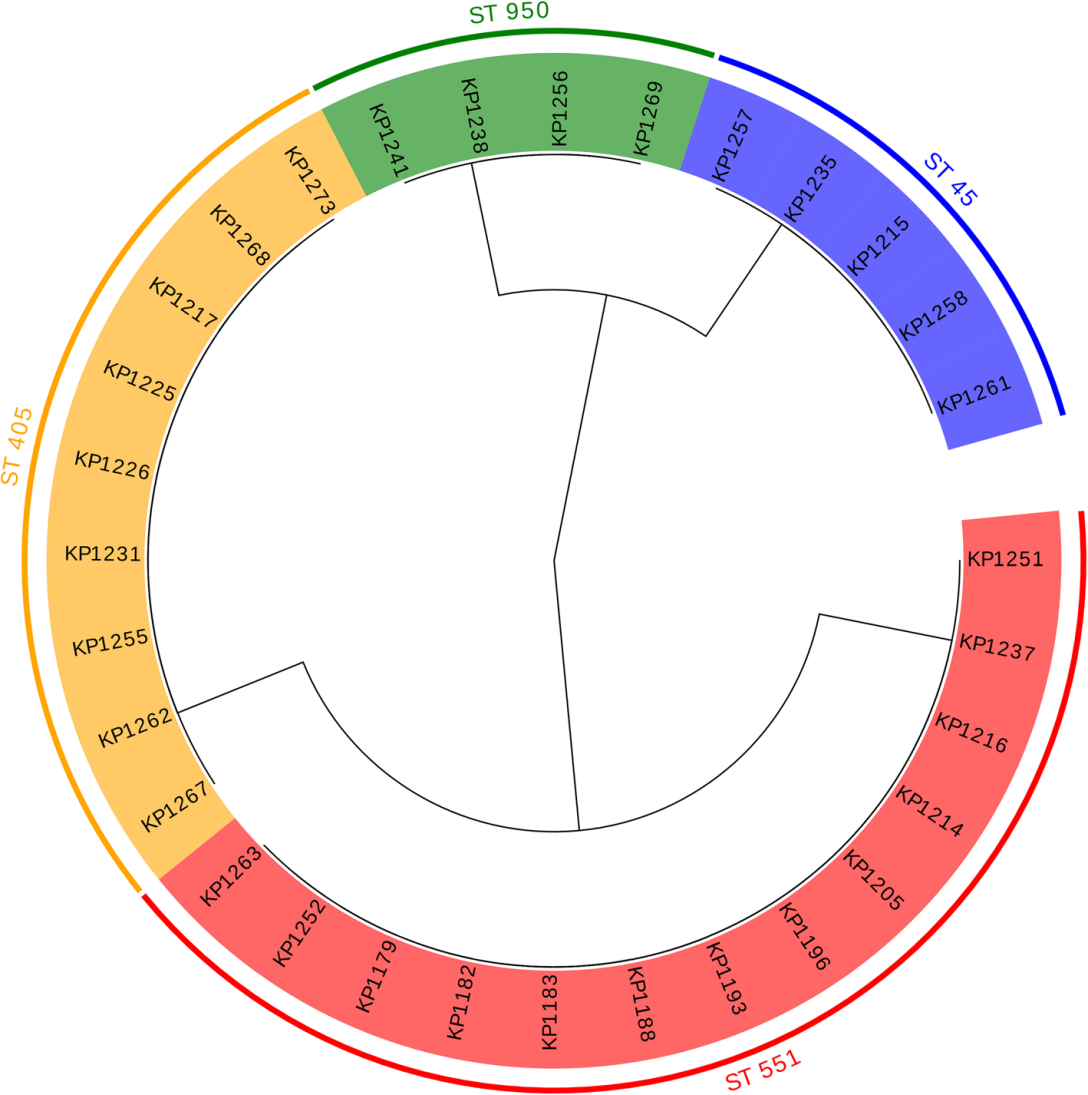
Cladogram of 31 *K. pneumoniae* isolates based on the variable fragment (KP_02) with highlight clusters obtained from MLST analysis created by Evolview [38]

For *K. pneumoniae* genomes, the identified fragments lengths were 12 bp (KP_02), 13 bp (KP_03), 21 bp (KP_04) and 26 bp (KP_05); thus, no erosion was needed. Again, the phylogenetic trees were constructed, and one of them created based on variable fragment KP_02 is depicted in Fig. 2. The other trees are shown in Additional file 1: Fig. S1 A-C.

All trees were constructed according to the process described in the section Phylogenetic analysis.

### Melting temperatures analysis

**Fig. 3:**
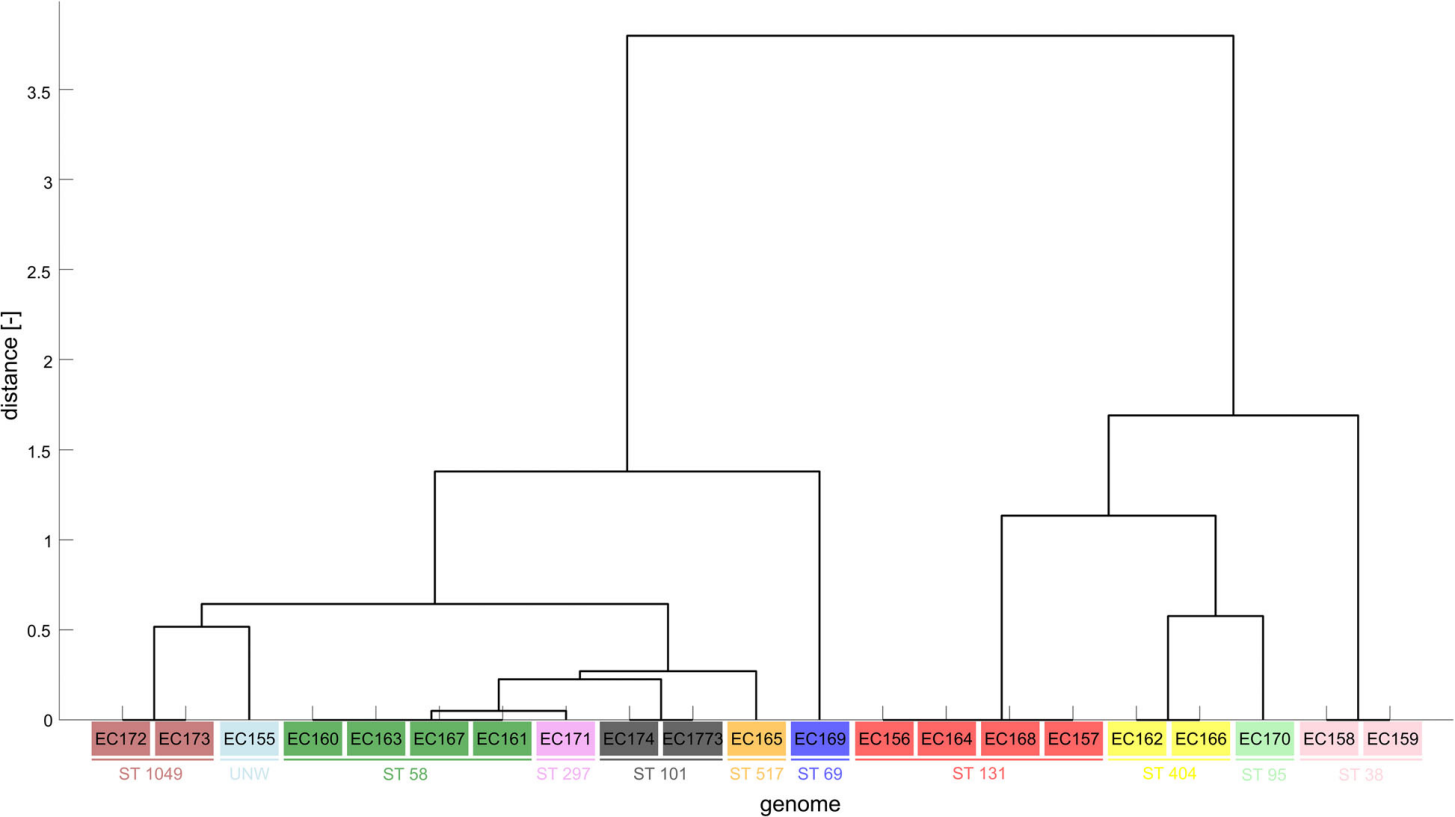
Dendrogram of 21 *E. coli* isolates obtained from cluster analysis of variable fragments (EC_01_1, EC_01_2, EC_01_3) melting temperatures with highlight clusters obtained from MLST analysis created

For *E. coli*, the melting temperatures for three variable fragments (EC_01_1, EC_01_2, EC_01_3) for each genome were calculated based on the nearest neighbor method [24] and are shown in the Table 6. A melting temperature cluster analysis was conducted, and the obtained dendrogram is depicted in Fig. 3.

**Table 6:** Calculated melting temperatures for each *E. coli* genome for three variable fragments

Genome ID	*T*_*m*_ of EC_01_1 variable fragment [ ^∘^C]	*T*_*m*_ of EC_01_2 variable fragment [ ^∘^C]	*T*_*m*_ of EC_01_3 variable fragment [ ^∘^C]
EC155	82.25	80.67	84.95
EC156	81.24	81.50	84.39
EC157	81.24	81.50	84.39
EC158	81.25	81.40	85.24
EC159	81.25	81.40	85.24
EC160	82.17	80.50	84.52
EC161	82.17	80.50	85.52
EC162	81.83	81.50	84.39
EC163	82.17	80.50	84.52
EC164	81.24	81.50	84.39
EC165	82.17	80.30	84.48
EC166	81.83	81.50	84.39
EC167	82.17	80.50	84.52
EC168	81.24	81.50	84.39
EC169	81.28	80.13	84.41
EC170	81.83	81.50	83.89
EC171	82.17	80.50	84.48
EC172	82.17	80.77	84.52
EC173	82.17	80.77	84.52
EC174	82.30	80.50	84.48
EC1773	82.30	80.50	84.48

**Fig. 4:**
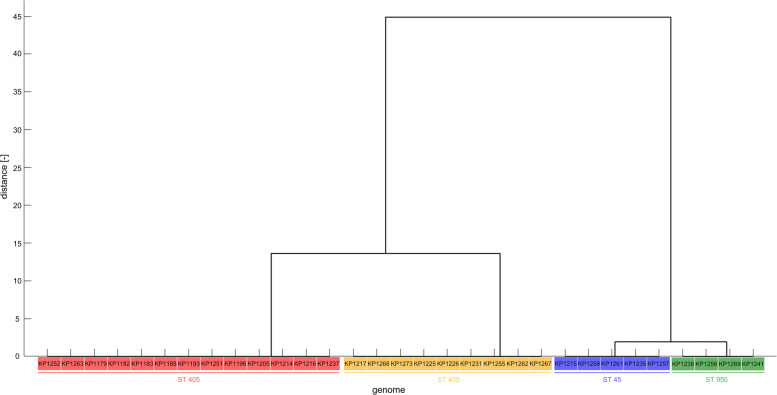
Dendrogram of 31 *K. pneumoniae* isolates obtained from cluster analysis of variable fragment (KP_02) melting temperatures with highlight clusters obtained from MLST analysis created

The melting temperatures were also calculated for variable *K. pneumoniae* isolate fragments, and cluster analysis was conducted. It was found that three (KP_02, KP_03, KP_05) out of four identified fragments could distinguish the genomes based on the calculated melting temperatures. The calculated values are shown in Table 7, and one obtained dendrogram based on fragment KP_02 is shown in Fig. 4. See other dendrograms in Additional file 1: Fig. S2 A-B.

**Table 7:** Calculated melting temperatures for each *K. pneumoniae* genome for three variable fragments

Genome ID	*T*_*m*_ of KP_02 variable fragment [ ^∘^C]	*T*_*m*_ of KP_03 variable fragment [ ^∘^C]	*T*_*m*_ of KP_05 variable fragment [ ^∘^C]
KP1179	33.92	37.20	63.37
KP1182	33.92	37.20	63.37
KP1183	33.92	37.20	63.37
KP1188	33.92	37.20	63.37
KP1193	33.92	37.20	63.37
KP1196	33.92	37.20	63.37
KP1205	33.92	37.20	63.37
KP1214	33.92	37.20	63.37
KP1215	44.36	46.40	60.98
KP1216	33.92	37.20	63.37
KP1217	29.74	36.46	62.24
KP1225	29.74	36.46	62.24
KP1226	29.74	36.46	62.24
KP1231	29.74	36.46	62.24
KP1235	44.36	46.40	60.98
KP1237	33.92	37.20	63.37
KP1238	45.28	44.98	60.70
KP1241	45.28	44.98	60.70
KP1251	33.92	37.20	63.37
KP1252	33.92	37.20	63.37
KP1255	29.74	36.46	62.24
KP1256	45.28	44.98	60.70
KP1257	44.36	46.40	60.98
KP1258	44.36	46.40	60.98
KP1261	44.36	46.40	60.98
KP1262	29.74	36.46	62.24
KP1263	33.92	37.20	63.37
KP1267	29.74	36.46	62.24
KP1268	29.74	36.46	62.24
KP1269	45.28	44.98	60.70
KP1273	29.74	36.46	62.24

The way the melting temperatures were determined is described in the Melting temperature calculation chapter.

The melting temperature calculation can be used to distinguish *E. coli* and *K. pneumoniae* strains. Thus, the fragments can be analyzed in mini-MLST. However, the difference in calculated temperature from different sequence types fluctuated only in the range of one degree is some cases. Therefore, the mini-MLST parameters, such as salt concentration, must be carefully determined.

## Conclusions

Bacterial genotyping is an essential process in epidemiology as it helps to find an infection’s source and monitor outbreaks. Results delivery should be done in the shortest possible time. However, typing methods are often laborious and computationally demanding, and financial costs are also high, especially for local clinical laboratories. The solution to the mentioned problems can be using mini-MLST, a cost-effective and efficient laboratory method.

The pipeline to identify variable sequences in the next-generation sequencing data that can be used in mini-MLST for bacterial typing was proposed and tested on 21 *E. coli* and 31 *K. pneumoniae* genomes. The hybrid assembly approach consists of reference-based mapping, and de novo assembly of unmapped reads was used. The preliminary location for variable fragments in the assembled scaffolds was carried out using variability rate calculation. In the selected fragments, the most variable parts were identified.

The melting temperatures were calculated to verify that the variable segments can be used in mini-MLST. The calculated melting temperature cluster analysis showed that distinguishing of individual strains is possible. Also, in contrast with MLST, mini-MLST does not use sequencing; thus, the bacterial genotyping cost will be significantly lower.

The proposed approach can be used to identify genomic regions that are not presented in the chosen reference sequence and can be specific to analyzed bacterial strains. Nevertheless, analyzing unmapped reads must be done carefully as the assembled sequences can contain parts of plasmids extracted with the genomic DNA.

One new variable fragment was located in *E. coli* isolates, and three variable fragments were identified in *K. pneumoniae* genomes. The identified fragments’ discriminatory power was the same as the seven housekeeping genes used in the MLST analysis. The most variable fragment regions were identified to ensure that it will be possible to perform mini-MLST. As only three regions located in the variable fragment in *E. coli* and one region in *K. pneumoniae* can be analyzed in mini-MLST instead of sequencing seven housekeeping genes, the analyzing time and cost will be significantly lower.

## Material and methods

### Dataset

The 21 *E. coli* and 31 *K. pneumoniae* isolates were collected in the Internal Hematology and Oncology Clinic at the University Hospital of Brno between 5/2019 and 7/2019. KAPA HyperPlus Kits (Roche, Switzerland) were used for sequencing library preparation, and a 2100 Bioanalyzer (Agilent Technologies, USA) was employed as a quality check. The prepared sequencing libraries were quantified with a KAPA Library Quantification kit (Roche, Switzerland) and the sequencing process using a MiSeq Reagent Kit v2 (500-cycles) was performed on an Illumina MiSeq platform. As a result, paired-end reads about 250 bp long were acquired.

### Reference-based assembly

Before genome assembly, the sequenced data quality was checked by FastQC (v0.11.5, [25]) combined with MultiQC (v1.7, [26]). BBMap (v38.71, [27]) software was used to map reads to the human genome (GRCh38.p13) to remove possible contamination. Then the Trimmomatic (v0.36, [28]) was employed for adapters and low quality read trimming. Reference-based mapping was applied, and NC_002695.2 [29] and NC_012731.1 [30] obtained from the RefSeq database [31] were chosen as the reference genomes for *E. coli* and *K. pneumoniae* assembly. The assembly was performed via Burrows-Wheeler Aligner MEM (v0.7.17-r1188, [32]).

### De novo assembly of unmapped reads

The reads that did not map to the reference genome were extracted, and the PCR and optical duplicated reads and low-quality reads were removed using Samtools (v1.9, [33]). In the next step, the St. Petersburg genome assembler (SPAdes) (v3.11.1, [34]) was employed for the unmapped reads’ de novo assembly. The assembly was run together with MismatchCorrector, and the Phred quality offset for the input reads was set to 33.

From genome assembly, the scaffolds were further analyzed. Due to a large number of short scaffolds, only those with a length greater than 500 bp were examined.

### Detection of variable regions

The variable fragments should be present in all isolates of the analyzed bacterium. For that reason, the scaffolds from one genome of *E. coli* (EC162) and one genome of *K. pneumoniae* (KP1241) were searched for in remaining genomes via BLAST+ (v2.6.0+, [35]).

**Fig. 5:**
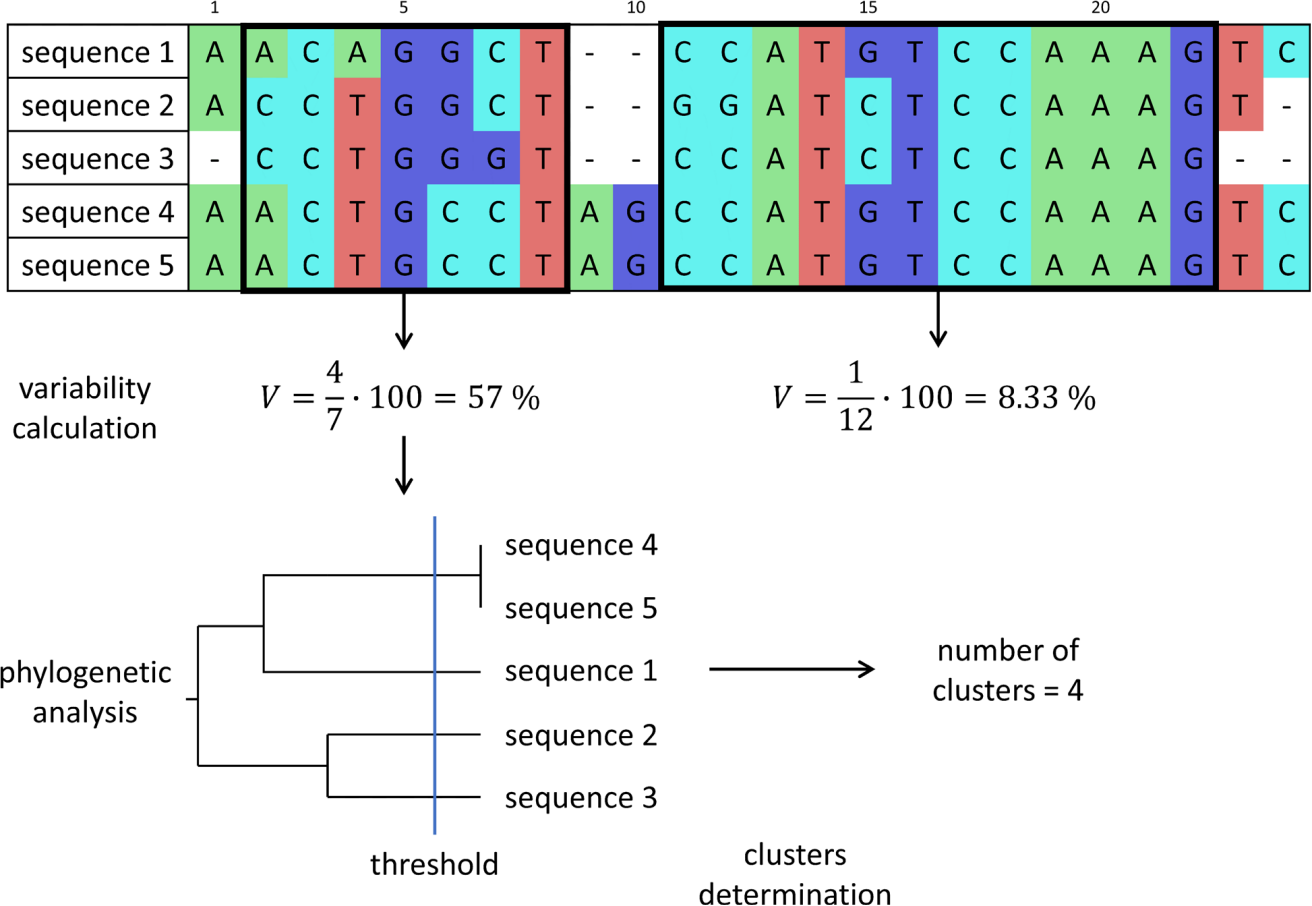
Scheme of variability regions’ calculation for a set of aligned sequences

The scaffolds that corresponded to the same region and were found in all genomes were aligned. From the alignment, the parts present in all genomes were analyzed. The variability rate was determined for each fragment of alignments. The variability *V* was calculated as

1 $$ V = \frac{n_{vp}}{n_{p}}\cdot 100,   $$

where *n*_*vp*_ is the number of variable positions in the alignment, and *n*_*p*_ is the number of all positions in the alignment. If the variability of an examined fragment is more than 10%, the fragment is further analyzed. The proposed process is shown in Fig. 5.

### Phylogenetic analysis

The evolutionary distances based on the Kimura [36] model were calculated for the segments of alignment with high variability. Using the distances, phylogenetic trees were constructed via UPGMA. Then a cluster analysis was conducted, and as a result, the number of clusters was calculated and compared with the number of sequence types obtained from the MLST analysis.

### Fragment shortening

If the variable fragment was too long to use in standard laboratory methods, it was shortened by two methods: erosion and dyadic decomposition. Firstly, erosion was performed. The nucleotides from the beginning of the sequence alignment were removed one by one. In each iteration, the phylogenetic tree was constructed, and controlling the number of clusters was performed to ensure that the discriminatory power was still preserved. As soon as the number of clusters decreased, the erosion was stopped. The same process was done from the end of the alignment. Secondly, fragment decomposition was carried out. As it was not possible to shorten the alignment using erosion, the fragment was split into two separate, equal halves. The first half of the alignment was shortened from the end as it was not possible to shorten it further from the beginning. For the second half of the alignment, the nucleotides were removed from the beginning. After each time it was shortened, the number of clusters was determined. If it was impossible to shorten the fragment further, each part was again split in the middle, and the process was repeated. Shortening was stopped when the length of the fragment was 120 bp.

### Melting temperature calculation

The melting temperatures were calculated for the variable fragments using the Oligo Calc [37]. The calculation parameters were left by default, and the melting temperatures computed using the nearest neighbor method were used for further analysis.

## Supplementary Information

**Additional file 1** Supplementary tables and figures: Table S1: MLST analysis results for 31 *K. pneumoniae* strains, Table S2: The identified variable fragments located in assembled scaffolds of *E. coli* genomes with number of clusters obtained by phylogenetic analysis, Table S3: The identified variable fragments located in assembled scaffolds of *K. pneumoniae* genomes with number of clusters obtained by phylogenetic analysis, Figure S1 A-C: Cladograms of 31 *K. pneumoniae* isolates based on variable fragments with highlight clusters obtained from MLST analysis, Figure S2 A-B: Dendrograms of 31 *K. pneumoniae* isolates obtained from cluster analysis of variable fragments melting temperatures with highlight clusters obtained from MLST analysis created.

## Data Availability

Raw sequencing data are available from the National Center for Biotechnology Information Sequence Read Archive database under a BioProject with accession number PRJNA695195 and PRJNA770840.

## Abbreviations

MLST: Multilocus sequence typing
mini-MLST: mini-multilocus sequence typing
ST: Sequence type
HRM: High resolution melting analysis
GC: guanine-cytosine
UPGMA: Unweighted pair group method with arithmetic mean
